# Comparison of Before and After Varicocelectomy Levels of Nitric Oxide in Seminal Fluid of Infertile Men

**DOI:** 10.5812/numonthly.4696

**Published:** 2012-09-24

**Authors:** Hossein Keyhan, Alireza Dadvar, Mohammad Ansari, Kheirollah Rafiee

**Affiliations:** 1 Urology Unit, Boali Hospital, Islamic Azad University Tehran Medical Branch, Tehran, IR Iran; 2 Department of Biochemistry, Tehran University of Medical Sciences, Tehran, IR Iran

**Keywords:** Varicocele, Nitric Oxide, Infertility, Semen

## Abstract

**Background:**

Since nitric oxide (NO) has an oxidant activity, lower levels following a varicocelectomy may result in better functioning sperm, improved semen quality and consequently higher fertility rates. However, this procedure should be examined in more detail.

**Objectives:**

Accordingly, this study was performed to compare the before and after varicocelectomy levels of NO in the seminal fluid of infertile men.

**Patients and Methods:**

In this before and after comparative study, 20 consecutive patients attending a training hospital in Tehran, Iran were recruited. All of these men had primary or secondary infertility accompanied with a varicocele. A semen sample was collected from the men in two phases, first before their varicocelectomy and two months after their operation.

**Results:**

NO levels differed significantly across the study and the mean (± standard deviation) levels of NO in the patients were 30.59 ± 10.35 µM/L and 21.48 ± 32.14 µM/L in the before and after phases of the study, respectively (P = 0.009).

**Conclusions:**

According to the results obtained in this study, it may be concluded that in future, levels of NO should be taken into consideration together with other parameters for the evaluation of patients who are affected by varicoceles, to determine probable therapeutic responses.

## 1. Background

A varicocele is a dilatation of the pampiniform venous plexus and the internal spermatic vein (1). Varicoceles are a well-recognized cause of decreased testicular function and they occur in approximately 15-20% of all males and in 40% of infertile males (2, 3). Varicoceles are the most common cause of poor sperm production and decreased semen quality (4). Varicoceles are easy to identify and to surgically correct (5, 6).

Reasons for surgical correction of a diagnosed varicocele include; relieving significant testicular discomfort or pain not responsive to routine symptomatic treatment, reducing testicular atrophy, and addressing the possible contribution to unexplained male infertility (7, 8). A varicocele may cause progressive damage to the testes, resulting in further atrophy and impairment of seminal parameters (2, 3).

A scrotal varicocele is the most correctable factor in a male with poor semen quality (4); therefore, varicocele repair should be considered a viable choice for appropriately selected individuals and couples with otherwise unexplained infertility. Varicocele repair has been shown to improve semen parameters in most men and creates possible improvements in fertility, in addition, the risks of varicocele repair are small (9-11). However, it is not clear what the leading cause of the therapeutic response in men with varicocele consists of, but it has been reported that nitric oxide levels in the seminal fluid are significantly different in men with a varicocele compared to those without this condition (12, 13).

Recent studies have shown that nitric oxide (NO) levels increase in the spermatic veins and seminal plasma of patients with varicoceles. Some observations have indicated that NO may modulate sperm function. Low concentrations of exogenous NO donors have been shown to enhance; human sperm motility, viability, capacitation, and binding to the zona pellucida (14-16). Conversely, at higher concentrations, they decrease human sperm motility and induce sperm toxicity (17, 18).

## 2. Objectives

Since nitric oxide has oxidization ability (19), this may decrease after a varicocelectomy resulting in better functioning of sperm and semen quality, with consequently higher fertility rates. However, this phenomenon should be examined in greater detail. Accordingly, this study was performed to compare the before and after varicocelectomy levels of nitric oxide in the seminal fluid of infertile men.

## 3. Patients and Methods

In this before and after comparative study, 20 consecutive patients attending a training hospital in Tehran, Iran were recruited. All of the men had primary or secondary infertility accompanied by varicoceles. The exclusion criteria were; history of nitrate drug use, active infection (especially urinary tract infection), and penile deformities leading to difficult insertion.

The semen samples were collected from the patients in two phases, first before the varicocelectomy and two months after the operation. The study was accepted by the Ethical Committee Board of the Tehran Branch of Azad University of Medical Sciences and was in accordance with good clinical practice and the Declaration of Helsinki. The study variables included; age, grade of varicocele, sperm count, normal morphology percentage, motility percentage, and level of NO in the seminal fluid.

Semen specimens were collected in sterile containers 48 to 72 hours after sexual abstinence. Specimens were allowed to thaw at room temperature for approximately 30 minutes, and a conventional semen examination was carried out under sterile conditions within one hour after collection. A part of the sample was stored at -20° C for a NO assay.

### 3.1. NO Assay

The thawed semen was centrifuged at 1 000 g for five minutes to remove the sperm cells. Then 50 µL of saturated zinc sulphate was added to 500 µL of each sample and left for 25-30 minutes at room temperature to precipitate the proteins. Thereafter, the samples were re-centrifuged with 13 000 g the supernatant which was collected for a nitrite assay (as an index of NO production) by the Griess method. In brief, one hundred µL of each sample were transferred to a 96-well microtiter plate and 100 µL of Griess reagent (2% sulfanilamide in 5% HCl solution and 0.1% N-(1-Naphtyl) ethylendiamine in water) was added. In addition, to reduce nitrate to nitrite, 100 µL vanadium chloride III 0.8% was added to each sample and they were left in an incubator at 37° C for one hour. The samples were then evaluated using a spectrophotometer method by an ELISA-reader (ANTHOS 2020, Anthos 2020 UK, Biochrom Ltd.) in 540 nm. Samples were run in triplicate to decrease the possibility of laboratory errors and finally the mean amounts were calculated. All chemicals and reagents were purchased from Merck, Germany.

The statistical analysis was performed using SPSS version 18.0 software (SPSS Inc, Chicago, IL, USA) using a Student’s t-test and Chi-square tests. A P value of 0.05 was considered significant.

## 4. Results

The mean (± standard deviation) age of the patients was 27.15 ± 4.79 years, ranging from 23 to 40 years. The varicocele grade was I in one patient (5%), II in 14 (70%), and III in five subjects (25%). There was no significant difference between sperm count, normal morphology percentage, and motility percentage among the patients in the before and after phases (Table 1), (P > 0.05).

**Table 1:** Spermogram Findings in Before and After Phases a

	**Phase**	**Mean**	**Standard Deviation**
**Count (x10 ^6^)**			
	Before	31.0692	31.22380
	After	31.8778	50.78838
**Motility (%)**			
	Before	40.77	18.010
	After	48.78	20.775
**Morphology (NL %)**			
	Before	62.31	18.085
	After	58.67	20.324

^a^There was no significant difference in any of the factors (P > 0.05).

NO levels differed significantly across the study and the mean (± standard deviation) levels of NO in the patients were 30.59 ± 10.35 µM/L and 21.48 ± 32.14 µM/L in the before and after phases of the study, respectively (P = 0.009). The age of the patients and the grade of the patients’ varicocele were not related to decreases in NO levels (P > 0.05). There was a significant linear association between an increase in sperm count, normal morphology percentage, and motility percentage with the amount of NO decrease (P < 0.05).

## 5. Discussion

NO production and consequently increased reactive oxygen free radicals may influence sperm production, motility and morphology in patients with varicoceles and result in poor fertility function (20-22). This situation is useful in explaining the pathogenesis of both testis and sperm dysfunction in varicoceles, especially since our study showed a noteworthy difference between NO levels in the before and after phases of this study and this decrease was significantly related to improved semen quality parameters.

Mitropoulos et al., (14) assessed peripheral blood samples in subfertile male subjects with varicoceles and compared them with blood samples from the dilated varicocele vein before ligation. The authors found elevated oxidative stress due to the release of nitric oxide synthase and xanthine oxidase within the dilated spermatic vein. This result was also observed in our study in the before-varicocelectomy phase.

In a similar study by Ozbek et al. (23) in Turkey, it was observed that preoperative and postoperative mean seminal fluid NO levels in patients with varicoceles were 114.82 +/- 33.02 µM/L and 93.17 +/- 27.24 µM/L, respectively showing a statistically significant difference between mean preoperative and postoperative seminal NO levels, this was similar to our study results.

Mostafa et al. (24) in Egypt found a statistically significant reduction in the three-month postoperative levels of NO compared with the pre-operative values in men who underwent a varicocelectomy, and this difference was observed even after one month. According to a study performed by Sakamoto et al. (25), there was a significant increase in sperm concentrations and reduction in NO levels following a varicocelectomy, this change was also observed in our study, but only for NO levels.

According to the results obtained in this study, it may be concluded that NO levels should be taken into consideration in the future, together with other parameters for the evaluation of patients who are affected by varicoceles to determine the probable therapeutic response. It may also be said, that patients with higher preoperative NO levels are possibly better candidates for a varicocelectomy and drug therapy, which will decrease NO levels in the seminal fluid and this may lead to better sperm quality.

However, further studies should be carried out to obtain more definite results, especially in larger sample populations. Observing patients’ seminal parameters (sperm count, motility and normal morphology) six months following a varicocelectomy to determine the best response to a varicocelectomy should also be included.
